# Association Between Cumulative Atherogenic Index of Plasma and New‐Onset Stroke Among Middle‐Aged and Elderly Chinese Patients With Stages 0–3 Cardiovascular‐Kidney‐Metabolic Syndrome: A Longitudinal Cohort Study

**DOI:** 10.1002/brb3.70914

**Published:** 2025-09-23

**Authors:** Miao Sun, Qingyu Yang, Ruonan Zhang, Xiaolin Zhang, Lisi Xu, Pengyu Pan

**Affiliations:** ^1^ Department of Neurology General Hospital of Northern Theater Command Shenyang China; ^2^ The 963rd Hospital of the PLA Joint Logistics Support Force Jiamusi China; ^3^ Department of the Second Cadre Ward General Hospital of Northern Theater Command Shenyang China; ^4^ Department of Neurology The First Affiliated Hospital of China Medical University Shenyang China; ^5^ Department of Neurosurgery General Hospital of Northern Theater Command Shenyang China

**Keywords:** atherogenic index of plasma, cardiovascular‐kidney‐metabolic, CHARLS, cumulative atherogenic index of plasma, stroke

## Abstract

**Background:**

Cardiovascular‐kidney‐metabolic (CKM) syndrome represents the interaction among chronic kidney disease, cardiovascular disease, and metabolic disorders. However, the association between the cumulative atherogenic index of plasma (CumAIP) and the risk of incident stroke in individuals with CKM syndrome has not been fully established.

**Methods:**

Data were obtained from the China Health and Retirement Longitudinal Study (CHARLS). A total of 4674 participants were categorized into quartiles according to CumAIP values and further stratified by CKM syndrome stages (0–3). Multivariable Cox regression models were applied to examine the relationship between CumAIP and incident stroke, while restricted cubic spline (RCS) models were used to explore potential nonlinear associations. Subgroup analyses evaluated possible effect modifications by age, sex, smoking, alcohol consumption, hypertension, and diabetes.

**Results:**

Over the follow‐up period, 261 (5.6%) incident strokes were documented, with incidence increasing from 2.5% in CKM stages 0 and 1 to 6.3% in stages 2 and 3. After adjusting for potential confounders, each unit increase in CumAIP was associated with a 149% higher risk of incident stroke (hazard ratio [HR] = 2.49, 95% confidence interval [CI]: 1.69–3.65). Participants in the highest CumAIP quartile showed a 144% higher risk compared with those in the lowest quartile (HR = 2.44, 95% CI: 1.61–3.70). RCS analysis suggested a linear association (*p* for nonlinearity = 0.182).

**Conclusion:**

Higher CumAIP is significantly associated with increased risk of incident stroke, particularly among individuals with advanced CKM stages and high‐risk subgroups, including older adults, smokers, and those with diabetes.

## Graphical abstract

Higher cumulative AIP is associated with an increased risk of new‐onset stroke among middle‐aged and elderly Chinese patients with CKM syndrome (stages 0–3).

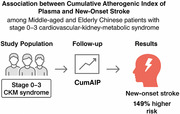

**Abbreviations**
AHA: American Heart Association
AIP: atherogenic index of plasma
ANOVA: analysis of variance
BMI: body mass index
CHARLS: China Health and Retirement Longitudinal Study
CI: confidence interval
CKD: chronic kidney disease
CKM: cardiovascular‐kidney‐metabolic
Cr: creatinine
CRP: C‐reactive protein
CumAIP: cumulative atherogenic index of plasma
CVD: cardiovascular disease
DBP: diastolic blood pressure
DM: diabetes mellitus
eGFR: Estimated Glomerular Filtration Rate
FBG: fasting blood glucose
HbA1c: glycated hemoglobin
HDL‐C: high‐density lipoprotein cholesterol
HR: hazard ratio
LDL‐C: low‐density lipoprotein cholesterol
RCS: restricted cubic spline
RFs: risk factors
SBP: systolic blood pressure
TC: total cholesterol
TG: triglyceride
UA: uric acid
WC: waist circumference

## 1 Introduction

Stroke remains a major global public health challenge (Feigin et al. 2021), with China contributing disproportionately to nearly one‐third of global stroke‐related mortality (S. Wu et al. 2019). Recent epidemiological estimates indicate that China experiences approximately 2.4 million incident strokes and 1.1 million stroke‐related deaths annually (Wang et al. 2017). The age‐standardized prevalence has reached 1114.8 per 100,000 population, with significantly higher incidence among middle‐aged and elderly individuals (S. Wu et al. 2019). Stroke has surpassed coronary heart disease as the leading cause of death in China (Zhou et al. 2016), placing a substantial burden on both healthcare systems and socioeconomic resources (L. Liu et al. 2011).

The cardiovascular‐kidney‐metabolic (CKM) syndrome provides an integrated pathophysiological framework that extends beyond conventional metabolic disorders to encompass cardiovascular and renal complications (Ndumele et al. 2023). This syndrome reflects a continuum initiated by metabolic dysfunction, driving systemic inflammation, endothelial dysfunction, and ultimately the development of atherosclerosis, collectively described as “pan‐vascular disease” (Larkin 2023). The progression of CKM highlights the intricate interplay between cardiovascular risk factors (RFs) and metabolic derangements, with hypertension, dyslipidemia, diabetes, and smoking identified as the most prevalent and modifiable contributors in China (Guan et al. 2017).

The atherogenic index of plasma (AIP), calculated as log (triglycerides [TGs]/high‐density lipoprotein cholesterol [HDL‐C]), has emerged as a valuable biomarker of both dyslipidemia and insulin resistance (Dobiásová and Frohlich 2001; Yin et al. 2023). Previous studies have identified AIP as a strong predictor of adverse cardiovascular outcomes (G. Cai et al. 2019; Qin and Chen 2024), with significant associations observed for intracranial arterial stenosis (Yu et al. 2024) and ischemic stroke (Y. Zhang et al. 2024). However, most investigations have relied on single‐time‐point measurements of AIP. In comparison, cumulative AIP (CumAIP) may better reflect the long‐term impact of lipid dysregulation on stroke risk (Z. Liu et al. 2024). Despite this, evidence exploring CumAIP about stroke among individuals with early to moderate CKM stages (0–3) remains scarce.

Since individuals with CKM stages 0–3 are at increased risk of progressive cardiovascular disease (CVD), clarifying the relationship between CumAIP and incident stroke in this group is essential. The China Health and Retirement Longitudinal Study (CHARLS) provides a unique opportunity to examine this association in a nationally representative cohort of middle‐aged and older Chinese adults (Zhao et al. 2014). Elucidating the role of CumAIP across CKM stages may inform targeted prevention strategies for those at greatest risk.

## 2 Materials and Methods

### 2.1 Research Design and Data Sources

This study used data from the CHARLS collected between 2011 and 2018. CHARLS is a nationally representative, open‐access, longitudinal cohort study designed to investigate the health, retirement, and socioeconomic status of Chinese residents aged 45 years and older. The baseline survey (Wave 1, conducted in 2011) employed a multistage, stratified probability sampling approach and recruited 17,708 participants from 28 provinces and 150 counties/districts across China. Subsequent follow‐up surveys were carried out biennially, including Wave 2 in 2013, Wave 3 in 2015, and Wave 4 in 2018.

### 2.2 Study Population

The initial study population consisted of 11,847 participants who underwent blood testing in the CHARLS baseline survey. A total of 7173 individuals were excluded based on the following criteria: (1) missing AIP‐related laboratory data (TGs and HDL‐C), (2) incomplete information on cardiovascular RFs (fasting blood glucose [FBG], glycated hemoglobin [HbA1c], body mass index [BMI], previous diagnoses of diabetes mellitus [DM] or hypertension, and smoking status), (3) follow‐up duration of less than 2 years or absence of TG and HDL‐C testing by the 2015 survey, (4) abnormal AIP values defined as lying outside the range of mean ± 3 standard deviations, (5) a documented history of stroke at baseline, (6) age younger than 45 years, and (7) diagnosis of CKM stage 4. After applying these exclusion criteria, 4674 participants were eligible and included in the final analysis (Figure S1).

Ethical approval for this study was obtained from Peking University (Approval number: IRB00001052‐11015), and all participants provided written informed consent. The data used in this study are publicly accessible through the official CHARLS website (http://charls.pku.edu.cn/en). Reporting of this research adhered to the guidelines of the Strengthening the Reporting of Observational Studies in Epidemiology (STROBE) statement (von Elm et al. 2008).

### 2.3 Exposure Assessment

Exposure was defined as the CumAIP. For each participant, the AIP was calculated as AIP = log10 (TG/HDL‐C)(Shi and Wen 2023), where TGs (mmol/L) and HDL‐C (mmol/L) were measured during each CHARLS survey using standardized laboratory procedures. To assess cumulative exposure over time, CumAIP was derived as CumAIP = [(AIP2012 + AIP2015)/2] × duration (2012–2015) (Zou et al. 2024). This approach has been widely applied in epidemiological studies to capture long‐term cumulative exposure of metabolic biomarkers (e.g., TgG‐BMI, TyG‐WHtR, and estimated glucose disposal rate) (Li et al. 2024; Ren et al. 2024; J. Zhang et al. 2025), thereby reducing the influence of short‐term fluctuations. We did not incorporate AIP values from 2018 because stroke events were ascertained between 2015 and 2018; inclusion of biomarker data measured after outcome assessment could introduce reverse causality due to disease onset or treatment modifications.

### 2.4 Outcome Assessment

The primary study endpoint was the occurrence of incident stroke during the follow‐up period spanning Waves 2–4. Consistent with previous research, stroke events were identified using standardized survey questions that collected self‐reported information on stroke history (Z. Zhang et al. 2023). Participants with a history of stroke at baseline were excluded from the analysis. The validity of stroke diagnoses within CHARLS has been confirmed in previous studies (Y. Wu et al. 2023).

### 2.5 CKM Evidence Stages 0–3

Participants were classified into CKM syndrome stages (0–3) based on the diagnostic framework outlined in the 2023 American Heart Association (AHA) Presidential Advisory Statement (Ndumele et al. 2023). The staging criteria were as follows:

1. *Stage 0 (no RFs for CKM)*: It includes normal BMI (<23 kg/m^2^ for Asians), normal waist circumference (WC; < 80 cm for women and < 90 cm for men), normal fasting glucose (< 5.6 mmol/L) or HbA1c (< 5.7%), normal lipid levels (TG < 150 mg/dL), normal blood pressure (systolic blood pressure [SBP] < 130 mmHg and diastolic blood pressure [DBP] < 80 mmHg), and absence of chronic kidney disease (CKD) or subclinical/CVD.
2. *Stage 1 (excessive or dysregulated adiposity)*: It includes BMI ≥ 23 kg/m^2^ (Asian standard) or WC ≥ 80 cm (women)/≥ 90 cm (men), without metabolic RFs (dyslipidemia and hypertension) or CKD.
3. *Stage 2 (metabolic RFs and CKD)*: It includes the presence of one or more metabolic RFs (hypertriglyceridemia, hypertension, DM, or metabolic syndrome) or CKD, defined as estimated glomerular filtration rate (eGFR) < 60 mL/min/1.73 m^2^ or evidence of proteinuria.
4. *Stage 3 (subclinical CVD)*: It includes the evidence of subclinical atherosclerosis (e.g., coronary artery calcification) or subclinical heart failure; or high 10‐year CVD risk, determined by the Framingham risk score, or substantially elevated CKD risk (CKD stage G4–G5 or KDIGO classification of substantially elevated risk).

The eGFR was calculated using the Chinese Modified Diet for Renal Disease (C‐MDRD) equation (Ma et al. 2006).

### 2.6 Data Collection

In line with previous studies, the following categories of data were collected:

1. *Demographic characteristics*: These include age, sex, marital status, educational attainment, and place of residence.
2. *Lifestyle factors*: These include smoking status and alcohol consumption.
3. *Physical measurements*: These include BMI, WC, SBP, and DBP.
4. *Medical history and medication use*: It includes previous diagnoses of hypertension, DM, or dyslipidemia, as well as the use of antihypertensive or glucose‐lowering medications.
5. *Laboratory tests*: These include TG, HDL‐C, low‐density lipoprotein cholesterol (LDL‐C), total cholesterol (TC), C‐reactive protein (CRP), HbA1c, creatinine (Cr), and uric acid (UA).

Participants were classified as hypertensive if they self‐reported a previous diagnosis of hypertension, reported use of antihypertensive therapy, or had an SBP ≥ 130 mmHg or DBP ≥ 80 mmHg at baseline. Similarly, participants were considered diabetic if they reported an earlier diagnosis of DM, were receiving glucose‐lowering treatment, or had FBG ≥ 7.0 mmol/L or HbA1c ≥6.5% at baseline.

### 2.7 Statistical Analysis

Based on the distribution of continuous variables (Y. Wu et al. 2023; Zou et al. 2024), normally distributed data were expressed as mean ± standard deviation, while non‐normally distributed data were presented as median with interquartile range. Between‐group differences were assessed using one‐way analysis of variance (ANOVA), the Kruskal–Wallis *H* test, or the chi‐square test, as appropriate.

To evaluate the association between CumAIP and incident stroke, multivariable Cox proportional hazards regression models were constructed. Four models were developed in a stepwise manner:

1. *Model 0*: unadjusted.
2. *Model 1*: adjusted for age and sex.
3. *Model 2*: further adjusted for educational attainment, marital status, residential area, smoking, alcohol consumption, and BMI.
4. *Model 3*: additionally adjusted for hypertension, diabetes, and dyslipidemia.
5. *Model 4*: further included laboratory covariates, specifically CRP and Cr.

Restricted cubic spline (RCS) regression was used to assess the dose–response relationship between CumAIP and incident stroke. To explore potential heterogeneity across CKM syndrome stages, subgroup analyses were performed separately for participants in CKM stages 0–3, with adjustments for all relevant covariates. Further stratified analyses were conducted to evaluate effect modification by age, sex, smoking status, alcohol intake, hypertension, and DM.

A two‐sided *p*‐value < 0.05 was considered statistically significant. All analyses were performed using R software, version 4.4.1.

## 3 Results

### 3.1 Baseline Features

A total of 4674 participants were included in the final analysis and stratified into quartiles according to CumAIP values (Table 1). The mean age of the study population was 58.1 ± 8.5 years, with a slightly higher proportion of women. Participants in higher CumAIP quartiles showed significantly elevated levels of TGs, TC, HbA1c, and hs‐CRP, while HDL‐C levels were significantly reduced. The prevalence of hypertension, DM, dyslipidemia, and higher BMI also increased progressively across CumAIP quartiles.

**TABLE 1:** Summary of baseline characteristics of the study population according to CumAIP quartile group.

Characteristic	Total (*n* = 4674)	Quartiles of the CumAIP				*p*‐value
		Q1 (−0.73 to 0.22)	Q2 (0.23–0.45)	Q3 (0.46–0.71)	Q4 (0.72–2.38)	
Age, years	58.1 ± 8.5	58.9 ± 8.8	58.3 ± 8.7	58.0 ± 8.3	57.3 ± 8.1	< 0.001
Gender, *n* (%)						< 0.001
Male	2125 (45.5)	613 (52.4)	516 (44.2)	500 (42.8)	496 (42.4)	
Female	2549 (54.5)	556 (47.6)	652 (55.8)	668 (57.2)	673 (57.6)	
Married, *n* (%)	4203 (89.9)	1045 (89.4)	1037 (88.8)	1047 (89.6)	1074 (91.9)	0.07
Education level, *n* (%)						0.01
Illiterate	1363 (29.2)	336 (28.7)	355 (30.4)	367 (31.4)	305 (26.1)	
Sishu/home school/elementary school	1936 (41.4)	504 (43.1)	505 (43.2)	438 (37.5)	489 (41.8)	
Middle school	950 (20.3)	232 (19.8)	214 (18.3)	245 (21)	259 (22.2)	
High school and above	425 (9.1)	97 (8.3)	94 (8)	118 (10.1)	116 (9.9)	
Family residence, *n* (%)						< 0.001
Urban	1490 (31.9)	298 (25.5)	357 (30.6)	391 (33.5)	444 (38)	
Rural	3184 (68.1)	871 (74.5)	811 (69.4)	777 (66.5)	725 (62)	
Smoking, *n* (%)						< 0.001
Never	2908 (62.2)	667 (57.1)	752 (64.4)	756 (64.7)	733 (62.7)	
Current and former	1766 (37.8)	502 (42.9)	416 (35.6)	412 (35.3)	436 (37.3)	
Drinking, *n* (%)						< 0.001
Never	2854 (61.1)	632 (54.2)	725 (62.1)	771 (66.1)	726 (62.1)	
Current and former	1817 (38.9)	535 (45.8)	443 (37.9)	396 (33.9)	443 (37.9)	
Hypertension, *n* (%)	1028 (22.0)	166 (14.2)	231 (19.8)	284 (24.3)	347 (29.7)	< 0.001
Diabetes, *n* (%)	235 (5.0)	33 (2.8)	38 (3.3)	74 (6.3)	90 (7.7)	< 0.001
Dyslipidemia, *n* (%)	359 (7.8)	37 (3.2)	75 (6.5)	100 (8.7)	147 (12.7)	< 0.001
BMI, Mean ± SD	23.5 ± 3.8	21.8 ± 3.1	23.0 ± 3.5	24.2 ± 4.0	25.2 ± 3.7	< 0.001
						
Cr, mg/dL	0.8 ± 0.2	0.8 ± 0.2	0.8 ± 0.2	0.8 ± 0.2	0.8 ± 0.2	0.009
HDL‐C, mg/dL	51.3 ± 15.3	66.3 ± 14.8	53.6 ± 10.5	47.6 ± 9.5	37.8 ± 9.3	< 0.001
LDL‐C, mg/dL	116.1 ± 34.3	110.9 ± 28.8	118.4 ± 32.2	122.3 ± 34.4	112.9 ± 39.8	< 0.001
TC, mg/dL	193.5 ± 37.9	188.6 ± 33.6	188.6 ± 36.1	194.3 ± 37.7	202.4 ± 42.1	< 0.001
UA, mg/dL	4.4 ± 1.2	4.2 ± 1.1	4.2 ± 1.2	4.4 ± 1.2	4.7 ± 1.3	< 0.001
HbA1c,%	5.3 ± 0.8	5.2 ± 0.6	5.2 ± 0.7	5.3 ± 0.7	5.4 ± 1.0	< 0.001
CRP, mg/L	1.0 (0.5, 2.0)	0.7 (0.4, 1.5)	0.8 (0.5, 1.7)	1.1 (0.6, 2.1)	1.3 (0.7, 2.5)	< 0.001
TG, mg/dL	104.4 (74.3, 153.1)	64.6 (53.1, 78.8)	89.4 (74.3, 108.9)	122.1 (99.1, 146.9)	200.9 (153.1, 275.2)	< 0.001
eGFR	110.6 ± 29.5	111.7 ± 26.8	112.3 ± 27.3	108.9 ± 28.0	109.4 ± 35.0	0.009
CumAip	0.5 ± 0.4	0.1 ± 0.1	0.3 ± 0.1	0.6 ± 0.1	1.0 ± 0.2	< 0.001
CKM stage, *n* (%)						< 0.001
0	198 (4.2)	103 (8.8)	67 (5.7)	23 (2)	5 (0.4)	
1	579 (12.4)	223 (19.1)	208 (17.8)	124 (10.6)	24 (2.1)	
2	1929 (41.3)	270 (23.1)	421 (36)	561 (48)	677 (57.9)	
3	1968 (42.1)	573 (49)	472 (40.4)	460 (39.4)	463 (39.6)	

*Note*: Values are presented as the mean (SD), median(quantile 1, quantile 3), or number (%), as appropriate.

Abbreviations: BMI, body mass index; Cr, creatinine; CRP, C‐reactive protein; CumAIP, cumulative atherogenic index of plasma; HbA1c, hemoglobin A1c; HDL‐C, high‐density lipoprotein‐cholesterol; LDL‐C, low‐density lipoprotein‐cholesterol; TC, total cholesterol; TG, triglycerides; UA, uric acid.

**FIGURE 1:**
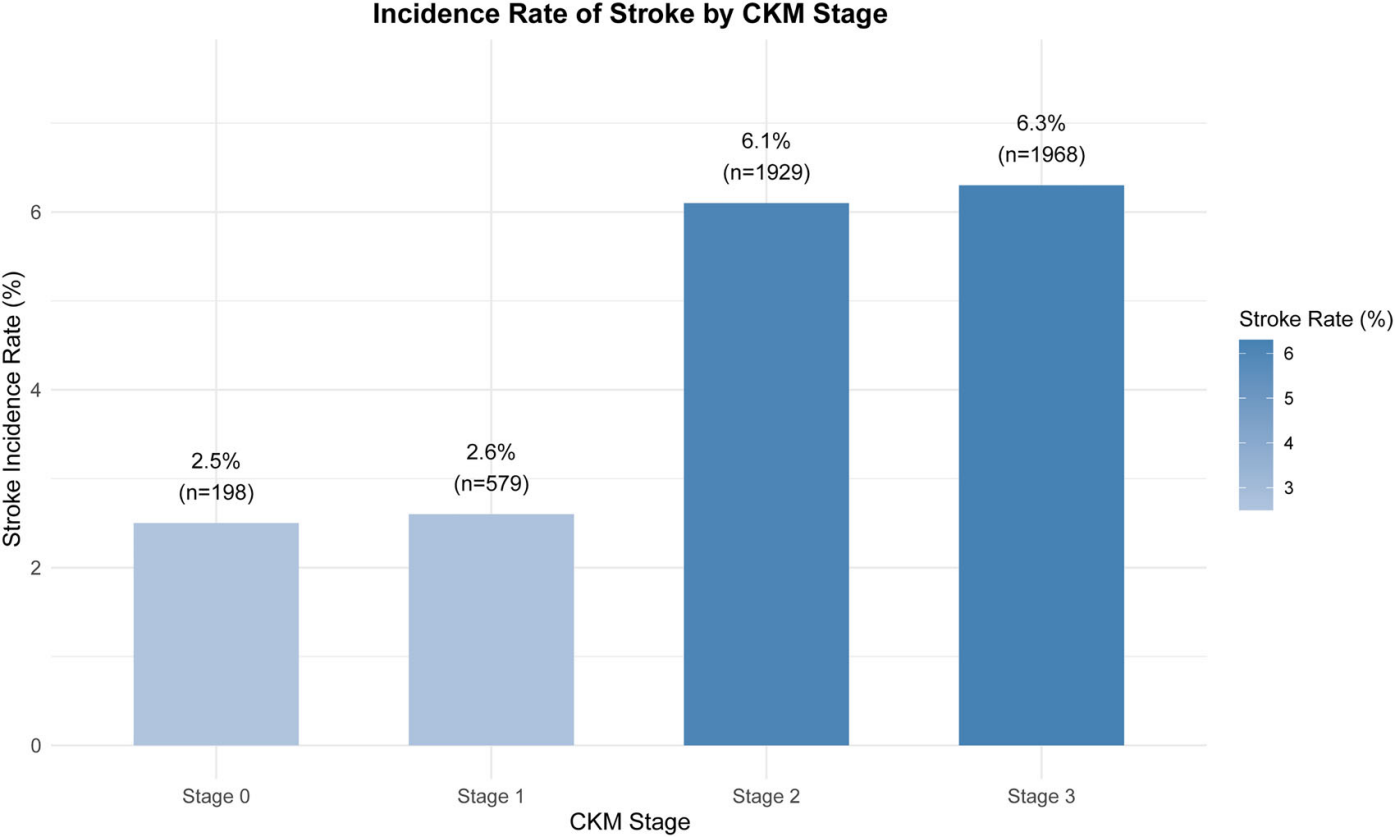
Incidence rate of stroke by CKM stage 0–3.

The incidence of incident stroke demonstrated a clear stage‐dependent trend with advancing CKM (Figure 1). Rates remained similar between stages 0 and 1, but rose sharply at stage 2 and subsequently plateaued at stage 3.

### 3.2 Correlation Between CumAIP and Stroke Incidence Among a CKM Syndrome Population (Stages 0–3)

Table 2 presents the association between CumAIP and incident stroke among participants with CKM syndrome stages 0–3. During the study period, 261 incident strokes (5.6%) were documented. The relationship between CumAIP and stroke risk was examined using five Cox proportional hazards models. In the unadjusted model, each one‐unit increase in CumAIP was associated with a 147% higher risk of incident stroke (hazard ratio [HR] = 2.47, 95% confidence interval [CI]: 1.84–3.32). After adjusting for age and sex (Model 1), the risk rose to 169% (HR = 2.69F, 95% CI: 2.00–3.61). Further adjustment for marital status, smoking, alcohol consumption, education, residential area, and BMI (Model 2) yielded a 156% increased risk (HR = 2.56, 95% CI: 1.88–3.50). With additional adjustments for hypertension, diabetes, and dyslipidemia (Model 3), the risk remained elevated by 132% (HR = 2.32, 95% CI: 1.68–3.18). The association persisted in the fully adjusted model (Model 4), which accounted for laboratory indicators including CRP and Cr, showing a 149% higher risk of incident stroke (HR = 2.49, 95% CI: 1.69–3.65).

**TABLE 2:** Association between the CumAIP and stroke incidence in a population with CKM syndrome stages 0–3.

Variable	Event%	Crude model	Model 1	Model 2	Model 3	Model 4
		HR (95%CI)	HR (95%CI)	HR (95%CI)	HR (95%CI)	HR (95%CI)
CumAIP	261 (5.6)	2.47 (1.84–3.32)	2.69 (2–3.61)	2.56 (1.88–3.5)	2.32 (1.68–3.18)	2.49 (1.69–3.65)
CumAIP (quartile)						
Q1	35 (3)	Ref.	Ref.	Ref.	Ref.	Ref.
Q2	62 (5.3)	1.8 (1.19–2.72)	1.88 (1.24–2.84)	1.84 (1.21–2.8)	1.76 (1.16–2.67)	1.8 (1.18–2.75)
Q3	66 (5.7)	1.91 (1.27–2.88)	2.03 (1.34–3.06)	1.96 (1.29–2.98)	1.82 (1.19–2.77)	1.82 (1.19–2.78)
Q4	98 (8.4)	2.86 (1.95–4.21)	3.14 (2.13–4.63)	2.92 (1.95–4.36)	2.61 (1.74–3.93)	2.44 (1.61–3.7)
*p*‐trend		< 0.001	< 0.001	< 0.001	< 0.001	< 0.001

*Note*: Model 1 includes age and gender. Model 2 includes age, gender, marital status, smoking, drinking, education level, family residence, and BMI. Model 3 includes age, gender, marital status, smoking, drinking, education level, family residence, BMI, hypertension, diabetes, and dyslipidemia. Model 4 includes age, gender, marital status, smoking, drinking, education level, family residence, BMI, hypertension, diabetes, dyslipidemia, CRP, Cr, TC, LDL‐C, UA, eGFR, and HbA1c.

To further evaluate this relationship, CumAIP was categorized into quartiles. Across all models, the association between higher CumAIP and incident stroke remained robust after sequential adjustment for demographic characteristics (Model 1), lifestyle factors (Model 2), underlying disease status (Model 3), and laboratory indicators (Model 4). In the fully adjusted model, participants in the highest CumAIP quartile (Q4) had a 144% greater risk of incident stroke compared with those in the lowest quartile (HR = 2.44, 95% CI: 1.61–3.70). Trend analysis demonstrated a significant dose–response relationship, with stroke risk increasing consistently across rising CumAIP quartiles (*p* for trend < 0.001).

**FIGURE 2:**
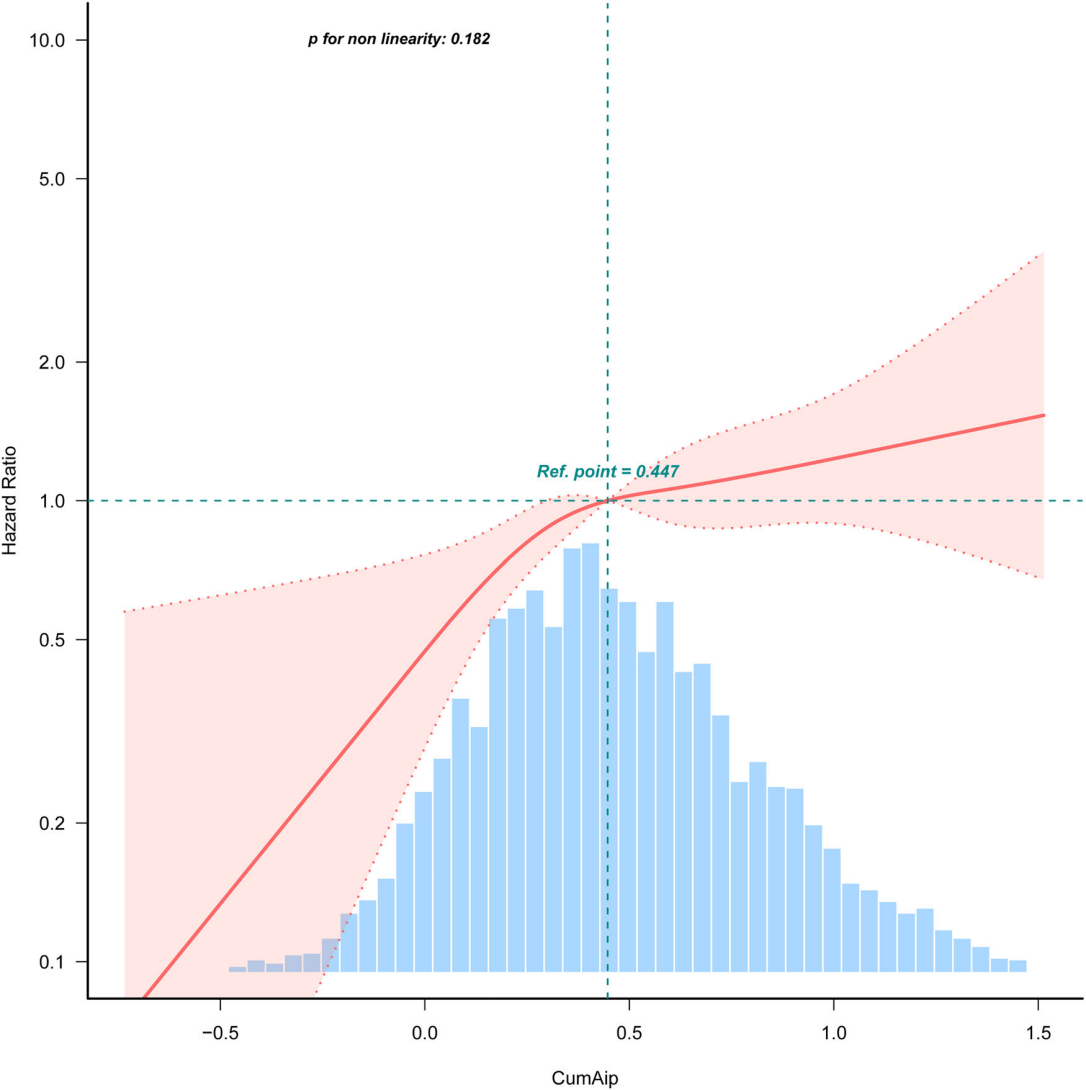
RCS analysis of the association between CumAIP and incident stroke among participants with CKM stages 0–3. Solid lines indicate predicted values, and dashed lines indicate 95% confidence intervals. The model was adjusted for age, gender, marital status, smoking, drinking, education level, family residence, BMI, hypertension, diabetes, dyslipidemia, CRP, Cr, TC, LDL‐C, UA, eGFR, and HbA1c.

The RCS analysis further confirmed the association between CumAIP and incident stroke across CKM stages 0–3, indicating a linear relationship (*p* for nonlinearity = 0.182) (Figure 2).

### 3.3 Subgroup Analyses

**FIGURE 3:**
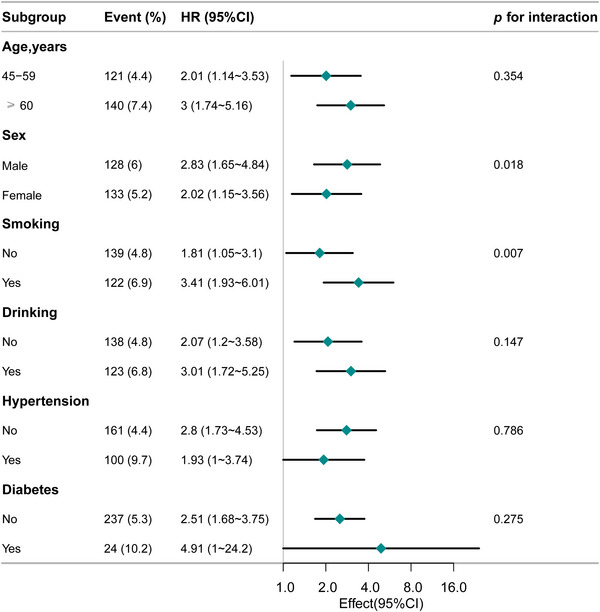
The relationship between CumAIP and stroke incidence according to basic features. Except for the stratification component itself, each stratification factor was adjusted for all other variables (age, gender, marital status, smoking, drinking, education level, family residence, BMI, hypertension, diabetes, dyslipidemia, CRP, Cr, TC, LDL‐C, UA, eGFR, and HbA1c).

Figure 3 illustrates the subgroup analysis of CumAIP and stroke risk. The association was particularly pronounced among participants aged 45–59 years (HR = 2.01, 95% CI: 1.14–3.53), ≥ 60 years (HR = 3.00, 95% CI: 1.74–5.16), males (HR = 2.83, 95% CI: 1.65–4.84), smokers (HR = 3.41, 95% CI: 1.93–6.01), alcohol consumers (HR = 3.01, 95% CI: 1.72–5.25), and individuals with diabetes (HR = 4.91, 95% CI: 1.00–24.2). Interaction tests indicated significant heterogeneity by sex and smoking status (*p* < 0.05). However, the consistent effect direction across subgroups and the possibility of chance findings due to multiple comparisons suggest that the relationship between CumAIP and stroke risk is broadly stable across different population subsets.

The association between CumAIP and stroke was further stratified by CKM stage (Table 3). The strongest effect was observed in participants with CKM stage 3 (*n* = 1968), where CumAIP was associated with a 183% higher risk of stroke after multivariable adjustment (HR = 2.83, 95% CI: 1.65–4.85, *p* < 0.001). Stage 0 also showed an extremely high HR (3091.58, 95% CI: 22.73–420,489.9, *p* = 0.001), though the wide CI indicates substantial uncertainty, likely reflecting the small sample size (*n* = 198, event rate 2.5%). For CKM stage 1 (HR = 5.23, 95% CI: 0.47–57.77, *p* = 0.177) and stage 2 (HR = 1.71, 95% CI: 0.94–3.09, *p* = 0.077), an elevated but nonsignificant risk was observed. No significant interaction was detected between CKM stage and CumAIP in predicting stroke (*p* for interaction = 0.219).

**TABLE 3:** Subgroup analysis of CumAIP and stroke incidence in CKM stages 0–3.

Subgroup	Number of participants	No. of Event (%)	Crude HR (95%CI)	*p‐*value	Adjusted HR (95%CI)	*p*‐value	*p* for interaction
CKM stage							
Stage 0	198	5 (2.5)	29.01 (0.94–891.11)	0.054	3091.58 (22.73–420489.9)	0.001	0.219
Stage 1	579	15 (2.6)	4.84 (0.57–41.22)	0.149	5.23 (0.47–57.77)	0.177	
Stage 2	1929	117 (6.1)	1.3 (0.8–2.11)	0.29	1.71 (0.94–3.09)	0.077	
Stage 3	1968	124 (6.3)	3.27 (2.17–4.93)	< 0.001	2.83 (1.65–4.85)	< 0.001	

The model was adjusted for age, gender, marital status, smoking, drinking, education level, Family residence, BMI, hypertension, diabetes, dyslipidemia, CRP, Cr, TC, LDL‐c, UA, eGFR, and HbA1c.

Furthermore, the RCS analysis confirmed a linear association between CumAIP and stroke risk among patients with CKM stage 3 (*p* for nonlinearity = 0.188) (Figure S2).

## 4 Discussion

To our knowledge, this study is the first to investigate the association between CumAIP and the risk of incident stroke among patients with CKM stages 0–3. We found that higher CumAIP levels were significantly associated with increased stroke risk, even after adjustment for multiple confounders, and this relationship remained robust across subgroup analyses. These results highlight CumAIP as a promising tool for stroke risk assessment and offer new perspectives for identifying high‐risk individuals and developing personalized prevention strategies.

Epidemiological evidence shows that nearly 90% of adults meet diagnostic criteria for CKM syndrome (stage 1 or higher), with about 15% at advanced stages (Aggarwal et al. 2024; Zhu et al. 2024). Consistent with this, in our cohort of 4674 participants, over 90% were classified as stage 1 or above, and stroke incidence increased with CKM progression from ∼2.5% in stages 0 and 1 to > 6% in stages 2 and 3. The pathogenesis of CKM begins with adipose tissue dysfunction (Ndumele et al. 2023). Excess adiposity promotes the secretion of proinflammatory cytokines (TNF‐α, IL‐6) and procoagulant factors (PAI‐1), while suppressing protective lipocalins, therefore driving systemic low‐grade inflammation (Hotamisligil 2006; Ouchi et al. 2011). Similarly, elevated free fatty acids disrupt insulin signaling and contribute to metabolic dysregulation (Hotamisligil 2017). Insulin resistance increases hepatic TG production, leading to dyslipidemia and endothelial dysfunction (Chakraborty et al. 2023). Hyperglycemia and hypertension further injure renal vasculature, causing glomerular hyperfiltration and eventually CKD (Seferović et al. 2018). In turn, metabolic waste accumulation and renin–angiotensin–aldosterone system (RAAS) activation exacerbate metabolic disturbances and cardiovascular injury (Marassi and Fadini 2023; Ndumele et al. 2023). This pathogenic framework underscores the urgent need for reliable biomarkers to improve stroke risk prediction in patients with CKM syndrome.

AIP has emerged as a valuable biomarker for assessing dyslipidemia, insulin resistance (Qu et al. 2024; Yin et al. 2023), and atherosclerotic lipid burden (Huang et al. 2023) as it reflects the ratio of TG to HDL‐C. Elevated AIP, indicative of dysfunctional lipid metabolism, contributes to stroke risk through several mechanisms. First, a high TG/low HDL‐C profile promotes the formation of small, dense LDL (sdLDL) particles and increases apolipoprotein B (ApoB) production, accelerating lipid deposition within the vascular wall (Dobiasova et al. 2011). Second, elevated TG induces oxidative stress in endothelial cells and suppresses nitric oxide (NO) synthesis, while reduced HDL‐C weakens anti‐inflammatory and endothelial‐protective functions, jointly exacerbating endothelial dysfunction (Chakraborty et al. 2023). Third, high TG activates toll‐like receptor (TLR) signaling, driving the release of inflammatory mediators such as CRP, IL‐6, and TNF‐α, which destabilize atherosclerotic plaques (M.‐Y. Wu et al. 2017). Finally, lipid abnormalities reflected by high AIP increase blood viscosity and platelet activity, elevate fibrinogen levels, and impair fibrinolysis, fostering a prothrombotic state (Fernández‐Macías et al. 2019). Together with impaired cerebrovascular autoregulation, these alterations significantly elevate ischemic stroke risk, particularly in individuals with comorbid hypertension or diabetes (Qu et al. 2024). Thus, AIP represents a cost‐effective and convenient predictor of incident stroke.

This study focused on CumAIP, which offers a more comprehensive measure of long‐term exposure to atherogenic lipid burden than single‐point AIP (Z. Liu et al. 2024; Zou et al. 2024). By incorporating the temporal dimension of lipid exposure (Zheng et al. 2023), CumAIP better reflects the cumulative balance between pro‐atherogenic and antiatherogenic lipoproteins (Dobiásová and Frohlich 2001; Won et al. 2021), while reducing the influence of short‐term fluctuations caused by diet, medications, or other transient factors (X. Cai et al. 2024; Zheng et al. 2023). This stability is crucial in patients with CKM syndrome, where recurrent metabolic disturbances and therapeutic interventions can significantly alter isolated lipid measurements.

This study observed a stepwise increase in stroke incidence with advancing CKM stage, with the highest risk noted in stage 3 patients (HR = 2.83, 95% CI: 1.65–4.85). In the fully adjusted model, each unit increase in CumAIP was associated with a 149% higher risk of stroke (HR = 2.49, 95% CI: 1.69–3.65). Similarly, individuals in the highest CumAIP quartile (Q4) had a 144% greater risk of stroke compared with those in the lowest quartile (Q1). These results are consistent with previous studies showing that atherogenic lipid abnormalities, particularly elevated TGs and reduced HDL‐C, are strongly associated with cardiovascular and cerebrovascular events (Esan and Wierzbicki 2021; Ference et al. 2019; Zheng et al. 2023). The RCS analysis further demonstrated a significant linear relationship between CumAIP and stroke risk (*p* for nonlinearity = 0.182), underscoring the potential of CumAIP as a continuous predictor and highlighting the clinical relevance of even modest increases in long‐term atherogenic burden.

Subgroup analyses further examined the association between CumAIP and new‐onset stroke across different populations. The relationship was particularly pronounced in older adults (≥ 60 years), smokers, and individuals with diabetes. In the elderly, the stronger association may reflect the cumulative impact of long‐term lipid dysregulation (Zeng et al. 2024), which accelerates atherosclerosis (Ali et al. 2024). This observation is consistent with findings from earlier large‐scale cohorts, such as one involving 9540 participants, which also demonstrated an increased stroke risk among older populations (Z. Liu et al. 2024). Among smokers, the elevated risk (HR = 3.41) suggests a synergistic effect between modifiable RFs and metabolic disturbances. The association was most evident in patients with diabetes (HR = 4.91, 95% CI: 1.00–24.2), likely reflecting more profound lipid metabolism abnormalities characterized by higher TG and lower HDL‐C levels (Chait et al. 2024), together with increased systemic inflammation (Libby 2012). In our cohort, participants with high CumAIP also showed elevated hs‐CRP, which may further impair endothelial function (Pourrajab et al. 2024). Moreover, diabetes‐related hypercoagulability, characterized by increased platelet activity and reduced fibrinolysis, may interact with elevated CumAIP to promote thrombosis and stroke (Kaur et al. 2018). Although risk magnitude varied across subgroups, interaction tests were not significant, indicating that CumAIP serves as an independent and broadly applicable predictor of stroke risk. These findings highlight the clinical value of CumAIP in identifying and managing high‐risk individuals across diverse populations.

This study has several significant advantages. To our knowledge, it is the first to investigate the association between CumAIP and incident stroke within a large, nationally representative longitudinal cohort of patients with CKM syndrome (stages 0–3). These findings demonstrate that higher CumAIP levels are linearly associated with an increased risk of stroke, supporting its potential as a practical, low‐cost, and accessible clinical marker for identifying high‐risk individuals.

However, certain limitations should be acknowledged. First, as an observational study, causal inferences cannot be established, and despite comprehensive adjustments for confounding factors, the possibility of residual confounding remains. Second, although stroke diagnosis in CHARLS was based on self‐report, this measure has been validated in previous studies and shown to be reasonably accurate for population‐based analyses (Ren et al. 2024). Nevertheless, information on ischemic versus hemorrhagic subtypes was unavailable. This is a limitation, as lipid‐related indices may have different associations with specific stroke subtypes. Third, information on lifestyle factors such as physical activity and dietary habits was not consistently available in CHARLS and therefore could not be adjusted for in our models. Fourth, the relatively small sample size in specific subgroups (e.g., CKM stage 0) reduced the precision of effect estimates. Finally, the study population consisted exclusively of middle‐aged and elderly Chinese adults. Given that lipid profiles and stroke risk are known to vary across ethnic groups—for instance, East Asians have relatively higher rates of hemorrhagic stroke compared to Western populations—the generalizability of our findings to other populations may be limited. Future studies in diverse multiethnic cohorts are needed to confirm these associations.

## 5 Conclusion

In conclusion, this study identified a clear linear relationship between higher CumAIP levels and an increased risk of new‐onset stroke among individuals with CKM syndrome stages 0–3. These findings highlight CumAIP as a simple, cost‐effective, and practical indicator for stratifying stroke risk in this population.

## Author Contributions

**Miao Sun**: writing – original draft, formal analysis, design. **Qingyu Yang**: statistical analysis, data collection, review. **Ruonan Zhang**: statistical analysis, data collection, review. **Xiaolin Zhang**: statistical analysis, data collection, review. **Lisi Xu**: methodology, validation, supervision, review. **Pengyu Pan**: methodology, validation, supervision, review. All authors collaborated on the article and cleared the presented version.

## Ethics Statement

Peking University provided the ethical approval for CHARLS (IRB00001052‐11015). All subjects and/or legal guardian(s) signed informed consent. All experiments were conducted according to the appropriate guidelines and regulations.

## Consent

The authors have nothing to report.

## Conflicts of Interest

The authors declare no conflicts of interest.

## Peer Review

The peer review history for this article is available at https://publons.com/publon/10.1002/brb3.70914.

## Supporting information




**Supplementary Figure 1**. Flow chart for the inclusion of participants in the study.

**Supplementary Figure 2**. RCS analysis of the association between CumAIP and incident stroke among participants with CKM stage 3. Solid lines indicate predicted values, and dashed lines indicate 95% confidence intervals. The model was adjusted for age, gender, marital status, smoking, drinking, education level, family residence, BMI, hypertension, diabetes, dyslipidemia, CRP, Cr, TC, LDL‐C, UA, eGFR, and HbA1.

## Data Availability

All employed data was sourced from the CHARLS website (http://charls.pku.edu.cn/).
