# Supplementary figures and images for: Association Between Cumulative Atherogenic Index of Plasma and New‐Onset Stroke Among Middle‐Aged and Elderly Chinese Patients With Stages 0–3 Cardiovascular‐Kidney‐Metabolic Syndrome: A Longitudinal Cohort Study
Source: Brain Behav. 2025 Sep 23;15(9):e70914. doi: 10.1002/brb3.70914 (PMC12457722; doi:10.1002/brb3.70914)

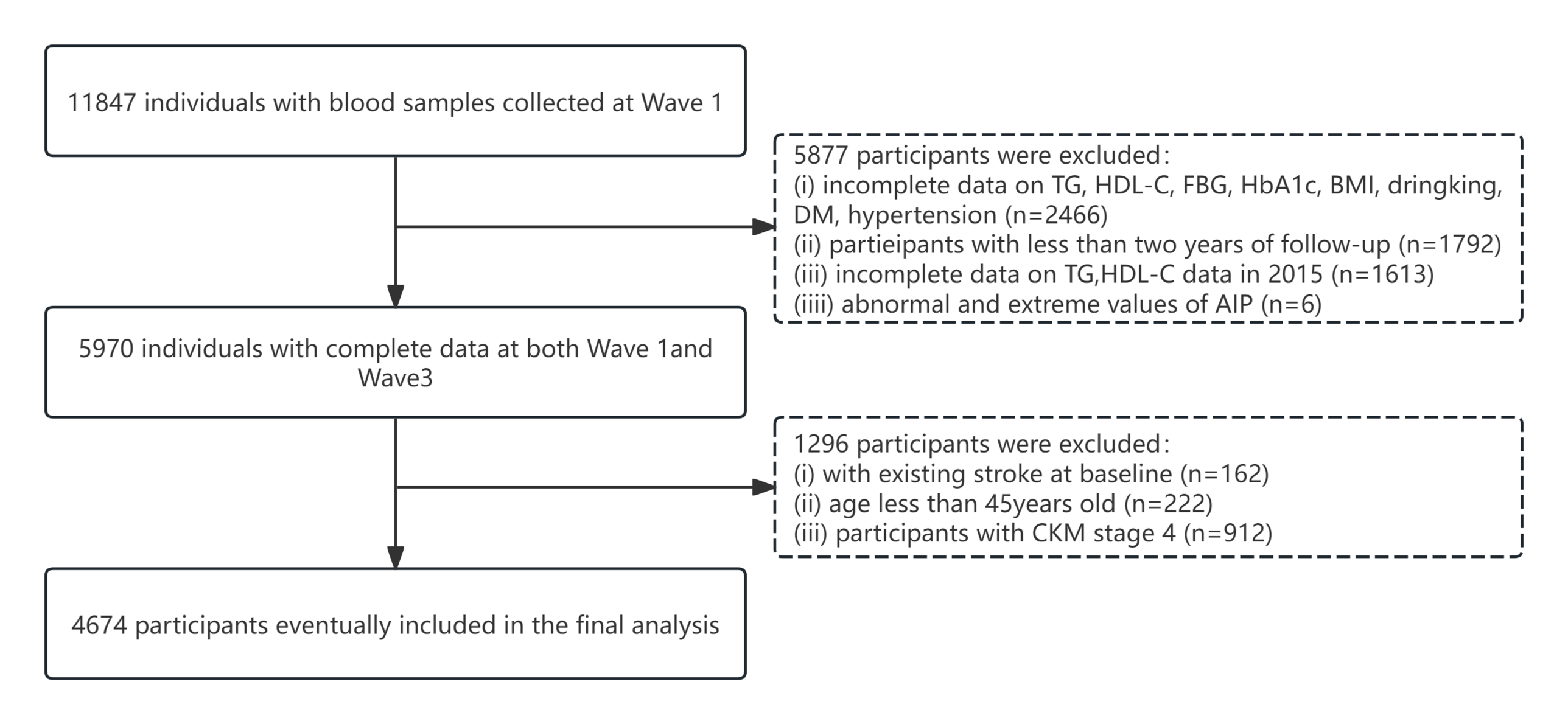

Supplement: Supplementary file 1 — Supplementary Figure 1. Flow chart for the inclusion of participants in the study. [file BRB3-15-e70914-s002.tif]

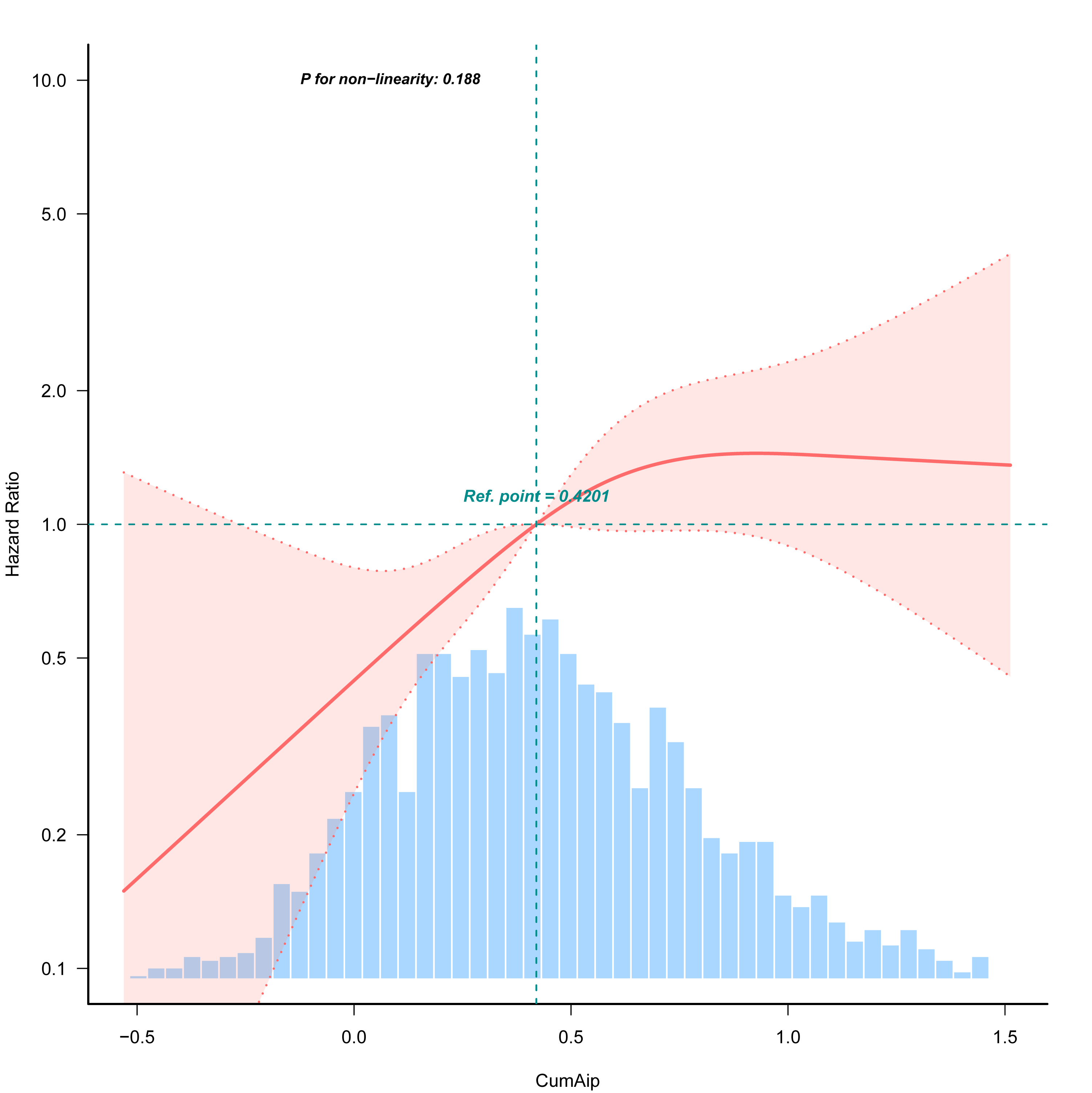

Supplement: Supplementary file 2 — Supplementary Figure 2. RCS analysis of the association between CumAIP and incident stroke among participants with CKM stage 3. Solid lines indicate predicted values, and dashed lines indicate 95% confidence intervals. The model was adjusted for age, gender, marital status, smoking, drinking, education level, family residence, BMI, hypertension, diabetes, dyslipidemia, CRP, Cr, TC, LDL‐C, UA, eGFR, and HbA1. [file BRB3-15-e70914-s001.tif]
